# 

**Published:** 1845-12-01

**Authors:** 


☞To Readers and Correspondents, &c. see cover, next page.

### TO READERS AND CORRESPONDENTS.

The whole of this number having been printed by the 18th ult. we are only able to acknowledge Exchange Periodicals, and other favors received, up to that date. For the same reason reference is not made to the contents of Periodicals already received, the Editor not having had time to give them proper examination. This remark applies particularly to the New-York Journal of Medicine, which contains an article by Dr. Dixon, relative to Ovarian Dropsy, to which allusion would have been made, had it been read before the Editor's remarks on the case furnished for this Journal were printed. The reader is referred to that article for some interesting cases of Ovarian Dropsy, cured by stimulating injections, and one case resembling that communicated by our correspondent.

### ACKNOWLEDGMENT OF PERIODICALS, &c.

☞ The following publications have been received up to Nov. 18th—

Boston Medical and Surgical Journal; Bulletin Medical Science for November; Transactions of the College of Physicians, Philadelphia, from May to October, 1845; New-York Medical Intelligencer; St. Louis Medical and Surgical Journal for Nov.; New-Orleans Medical and Surgical Journal for Nov.; Ninth Annual Report of the Trustees and Superintendent of the Vermont Asylum for the Insane, Sept. 1845, from Dr. Rockwell, the Medical Superintendent; New-York Medical and Surgical Reporter, Nos. 2 and 3.

### BUFFALO MEDICAL ASSOCIATION.

The meeting of this Association for December, will be holden at the office of Drs. B. and G. N. Burwell, on the evening of the 2d inst. at 7 o'clock.

AUSTIN FLINT, Rec. Secretary.

☞ Books, Pamphlets, Magazines, &c. &c. rebound in the neatest manner. Particular care taken in arranging the plates, &c. of Periodicals.

☞ Mr. T. S. CUTTING is authorized to act as Agent for this Journal, collect subscriptions, &c.

